# Supplementary material for: Identification of photoperiod-induced specific miRNAs in the adrenal glands of Sunite sheep (Ovis aries)
Source: Front Vet Sci. 2022 Jul 22;9:888207. doi: 10.3389/fvets.2022.888207 (PMC9354845; doi:10.3389/fvets.2022.888207)
Supplement: Supplementary file 1 [file Data_Sheet_1.ZIP › Supplementary file/miRDeep2 file.zip!/miRDeep2 file/Know_Structure/SPLP42A3/oar-mir-221.pdf]

miRBase precursor : oar-mir-221
Total read count : 1113
oar-miR-221 read count : 875
remaining reads : 238

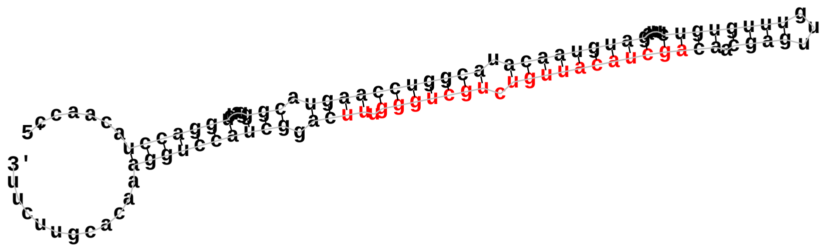

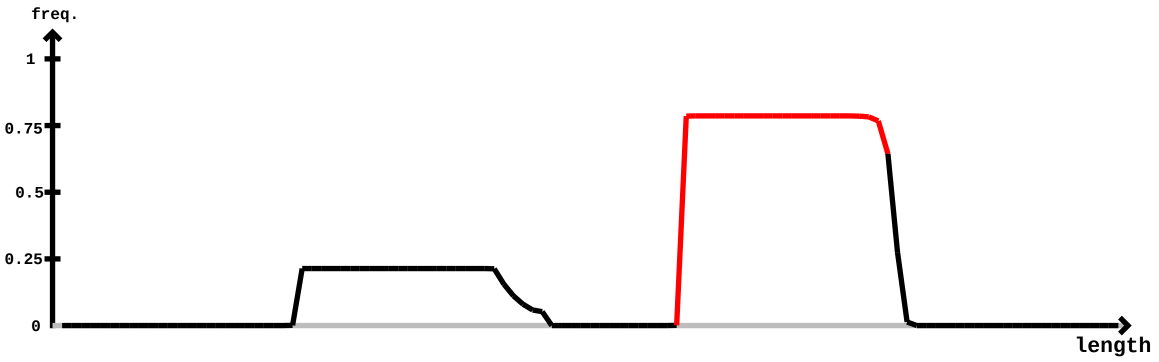

oar-miR-221

```
5'- ccaacauccaggucuagggcaugaaccuggcauacaauguagauuucuguguuuguugagcaacagcuacauugucugcuggguuucaggcuaccuggaaacacguucuu   -3'    exp
    ......(((((((....(((.(((((((((((.(((((((((....((((((((...)))).))))))))))))).))))))).)))).))))))))))...........   reads   mm   sample
    .......................aaccuggcauacaauguagau..................................................................   1       0    seq
    ........................accuggcauacaauguagauu.................................................................   64      0    seq
    ........................accuggcauacaauguagauuu................................................................   49      0    seq
    ........................accuggcauacaauguagauuuc...............................................................   35      0    seq
    ........................accuggcauacaauguagauuucu..............................................................   24      0    seq
    ........................accuggcauacaauguagauuucug.............................................................   7       0    seq
    ........................accuggcauacaauguagauuucugu............................................................   58      0    seq
    ...............................................................cagcuacauugucugcuggguuu........................   1       0    seq
    ................................................................agcuacauugucugcugg............................   1       0    seq
    ................................................................agcuacauugucugcuggg...........................   3       0    seq
    ................................................................agcuacauugucugcuggggu..........................   17      0    seq
    ................................................................agcuacauugucugcuggguu.........................   136     0    seq
    ................................................................agcuacauugucugcuggguuu........................   410     0    seq
    ................................................................agcuacauugucugcuggguuuc.......................   291     0    seq
    ................................................................agcuacauugucugcuggguuuca......................   15      0    seq
    .................................................................gcuacauugucugcuggguu.........................   1       0    seq
```
